# When less may be more: calorie restriction and response to cancer therapy

**DOI:** 10.1186/s12916-017-0873-x

**Published:** 2017-05-24

**Authors:** Ciara H. O’Flanagan, Laura A. Smith, Shannon B. McDonell, Stephen D. Hursting

**Affiliations:** 1 Department of Nutrition, University of North Carolina, Chapel Hill, NC 27517 USA; 2 Lineberger Comprehensive Cancer Center, University of North Carolina, Chapel Hill, NC 27517 USA; 3 Nutrition Research Institute, University of North Carolina, Kannapolis, NC 28081 USA; 4 Department of Nutrition, University of North Carolina at Chapel Hill, 2100 Michael Hooker Research Center, Campus Box 7461, Chapel Hill, NC 27599 USA

**Keywords:** Calorie restriction, Chemotherapy, Radiation therapy, Ketogenic diet, Fasting, Metabolism, Autophagy, Cachexia, Insulin-like growth factor 1, Drug resistance

## Abstract

Calorie restriction (CR) extends lifespan and has been shown to reduce age-related diseases including cancer, diabetes, and cardiovascular and neurodegenerative diseases in experimental models. Recent translational studies have tested the potential of CR or CR mimetics as adjuvant therapies to enhance the efficacy of chemotherapy, radiation therapy, and novel immunotherapies. Chronic CR is challenging to employ in cancer patients, and therefore intermittent fasting, CR mimetic drugs, or alternative diets (such as a ketogenic diet), may be more suitable. Intermittent fasting has been shown to enhance treatment with both chemotherapy and radiation therapy. CR and fasting elicit different responses in normal and cancer cells, and reduce certain side effects of cytotoxic therapy. Findings from preclinical studies of CR mimetic drugs and other dietary interventions, such as the ketogenic diet, are promising for improving the efficacy of anticancer therapies and reducing the side effects of cytotoxic treatments. Current and future clinical studies will inform on which cancers, and at which stage of the cancer process, CR, fasting, or CR mimetic regimens will prove most effective.

## Background

### Calorie restriction (CR) and cancer

CR, a chronic reduction of dietary energy intake by approximately 30% without incurrence of malnutrition, is a broadly effective dietary intervention that significantly decreases adiposity and inflammation and improves metabolic profiles in non-obese humans and rodents [1–4]. Preclinical studies in mammalian models demonstrate that CR extends lifespan, ameliorates risk factors, and delays onset of age-related diseases, including cancer, type II diabetes, and cardiovascular and neurodegenerative diseases [4]. In response to decreased caloric intake, metabolic alterations foster health promoting characteristics, including increased insulin sensitivity and decreased blood glucose, growth factor signaling, inflammation, and angiogenesis [4]. While the impact of CR on age-related pathologies has been studied most extensively in rodent models, data from human observational and randomized clinical trials demonstrate that CR in non-obese humans results in metabolic and molecular changes similar to those observed in rodent models [5]. Within the scope of cancer research, a meta-analysis of preclinical rodent models evaluated the impact of CR across multiple cancer types and through a variety of tumor models [6]; overall, CR displayed a 75.5% reduction in tumor incidence. Longitudinal studies at the National Institute of Ageing and the University of Wisconsin showed a significant reduction in the incidence of cancers in rhesus monkeys fed a CR diet compared to a control diet [7]. While the antitumorigenic effects of CR are well established, the mechanism behind this relationship remains unclear, though it is believed that the tumor suppressive effects are mediated, in part, by enhanced apoptosis within tumors, modulation of systemic signals such as insulin-like growth factor (IGF)-1, insulin, metabolic and inflammatory pathways, as well as by reduced angiogenesis.

Exposure to an energy restricted diet results in reduced systemic glucose and growth factors such as IGF-1 [1, 8, 9]. Preclinical studies in breast, pancreatic, and colon cancer have demonstrated that modulation of IGF-1 signaling plays a major role in CR’s anticancer effects [8, 10, 11]. In alignment with this, population studies have demonstrated that the IGF-1 signaling pathway plays a significant role in the development and progression of many cancer types [12]. IGF-1 is a nutrient-responsive growth factor that activates two major signaling cascades, namely Ras/MAPK and PI3K/AKT. Activation of the Ras/MAPK pathway promotes activity of transcription factors and subsequent expression of genes involved in proliferation and cellular growth. Initiation of the PI3K/AKT pathway promotes decreased apoptosis by disrupting the BCL2-Bad complex, increases protein synthesis via mTOR activation, and increases glucose metabolism by inhibiting GSK-3β [13]. Cancer cells utilize the IGF-1 signaling pathway to redirect their metabolic investment towards proliferation and growth, and thus reduction of IGF-1 levels in CR results in decreased tumor growth and progression [8, 10, 11]. Addition of exogenous IGF-1 leads to the partial reversal of the anticancer effects of CR, further supporting the role of IGF-1 in tumorigenesis [10]. In addition, expression of signaling factors downstream of IGF-1 has been correlated with either resistance or sensitivity to several cancer therapies [14]. Nevertheless, although IGF-1 signaling is a promising anticancer target, drugs targeting the pathway have been largely unsuccessful [12].

CR also induces activation of AMP-activated protein kinase (AMPK), a molecular sensor that increases catabolism and inhibits anabolic metabolism, working in opposition to IGF-1-mediated activation of mTOR [4, 15]. AMPK activation in response to CR conditions results in increased apoptosis within brain tumors while protecting normal cells from the stress [16, 17]. AMPK induces expression of metabolic control genes, including SIRT1, resulting in increased fatty acid oxidation and glutaminolysis to provide auxiliary substrates when glucose is scarce [18]. In line with this, CR results in elevated serum glutamine [19] and ketone bodies [20]. Many tumors undergo metabolic reprogramming, including enhanced fatty acid oxidation and glutaminolysis in addition to increased glucose metabolism [21–23]. Some cancers can therefore become autonomous, uncoupling their growth from the availability of systemic factors under normal conditions. Despite it not being clear whether administration of CR would support tumor growth in these circumstances, it is thought that transformed cells lack the metabolic adaptability to respond to altered substrate availability, having already invested heavily in metabolic reprograming and thus being more sensitive to increased pressure on ketone metabolism and fatty acid oxidation than normal cells [24].

The AMPK/mTOR axis also controls autophagy, a process through which proteins, macronutrients and organelles are enveloped in double-membraned vesicles and degraded into building blocks that can then be shuttled into synthetic pathways. Under CR conditions, AMPK activation stimulates increased autophagy to recycle cellular components and provide needed substrates for metabolism and homeostasis [4, 15]. Autophagy can act as a double-edged sword in cancer – it is thought to be tumor suppressive since defects in autophagy drive oxidative stress, mitochondrial defects, DNA damage, genomic instability, and tumor growth [25]. Conversely, it is believed to be tumor promoting because established tumors can utilize autophagy to reduce oxidative stress and increase mitochondrial function and metabolism in order to promote survival and overcome stress and low nutrient conditions [25, 26]. Due to the dual role of autophagy in cancer, autophagy inducers and inhibitors have become targets for cancer therapy [27]. Recent research in our lab showed that combining autophagy inhibition with a CR regimen reduced tumor growth more than either treatment alone [20].

Furthermore, increasing preclinical and human evidence suggests that CR reduces inflammation [11, 28, 29]. Multiple inflammatory signaling cascades can promote the growth and survival of neoplastic cells [30]. The reduction of energy intake in CR reduces the amount of adipose tissue, a major endocrine organ that secretes pro-inflammatory factors including leptin, adiponectin, monocyte chemo-attractant protein-1, tumor necrosis factor, and interleukin-6 [31]. CR in human studies is associated with reduced adiposity and a lessened inflammatory adipose secretome, as well as particularly decreased systemic levels of pro-inflammatory adipokines [32]. In addition, CR has been shown to consistently reduce the expression of the pro-angiogenic factors vascular endothelial factor [9, 33] and plasminogen activator inhibitor-1 [15], both of which induce the growth of new blood vessels to provide growing tumors with oxygen and glucose. CR has also been shown to reduce vascularization of tumors [9, 16]. In addition to altering systemic inflammatory mediators, CR has been shown reduce the expression of inflammatory genes in cancer cells, including nuclear factor kappa B [11, 34] and peroxisome proliferator-activated receptors [35], which are ligand-activated transcription factors involved in regulating inflammation, proliferation, and glucose and lipid homeostasis and often expressed in cancer cells [36, 37].

### CR and therapeutic response

To date, much of the research into the tumor suppressive effects of CR relate to the preventative capacity of the intervention, rather than its application as an anticancer therapy. Recent attention has focused on the potential of CR as an adjunct therapy for a range of cancers in combination with traditional chemotherapy or radiation therapy [38]. While chronic CR may be well tolerated in preclinical and clinical studies of healthy individuals, patients diagnosed with cancer are at a greater risk of weight loss due to toxic cancer therapies, as well as cachexia and sarcopenia from tumor-derived signals to degrade adipose and muscle tissues, to which chronic CR may contribute. Furthermore, as CR is anti-inflammatory, chronic CR may be a concern to patients with immunodeficiency or following surgery. Intermittent CR, achieved through fasting where no calories are consumed for defined periods of time (between 24 h up to 6 days), causes similar metabolic and anti-inflammatory alterations as seen during chronic CR, and can often result in greater changes in the short term [39]. In contrast to chronic CR, fasting results in glycogen release from the liver for use as an energy source. Once glycogen stores are depleted, amino acids and fatty acids are catabolized to generate glucose and ketone bodies, respectively [39]. Short-term fasting has been shown to improve chemotherapeutic treatment with etoposide [40], mitoxantrone, oxaliplatin [41], cisplatin, cyclophosphamide, and doxorubicin [42] in transgenic and transplant mouse models of neuroblastoma, fibrosarcoma, glioma, melanoma, and breast and ovarian cancers. Alternate day fasting has also been shown to improve the radiosensitivity of mammary tumors in mice [38, 43], likely due to enhanced oxidative stress and DNA damage during short-term fasting on cancer cells. Fasting has also been shown to control circadian clock genes, the expression of which usually oscillates at specific intervals throughout the day and is coupled to processes such as oxidative stress response and DNA damage repair [44]. Chemotherapeutic treatment administered at different times of the day has been found to improve efficacy, believed to be due to circadian rhythmic control of stress responses [45]. Fasting may therefore improve the efficacy of anti-cancer therapies in part by controlling the circadian rhythm.

Elegant work by Rafaghello et al. [40] demonstrated that short-term fasting elicits differential responses to chemotherapy in normal and cancer cells, with normal cells inactivating growth signals, such as Ras, Akt and IGF-1, in response to short-term fasting protecting them from therapeutic toxicity. In contrast, cancer cells, which have arisen due to activation of these signals and evasion of senescence-inducing signals, do not undergo this inactivation, remaining vulnerable to cytotoxic treatment via chemotherapy or radiation therapy. The induction of these cancer-specific stress responses may impact mechanisms related to chemoresistance, including multidrug resistance [46].

More recently, a fasting-mimicking diet, in which mice are fed the same amount of food as control mice, albeit with a severely reduced caloric density, showed a similar reduction in tumor growth as short-term starvation and displayed synergistic therapeutic effects when combined with doxorubicin and cyclophosphamide [41, 47]. Cycles of this diet have recently been shown to improve metabolic and inflammatory biomarkers associated with cancer risk in humans [48]. Mechanistically, the synergizing effects of the fasting-mimicking diet were associated with increased autophagy in the cancer cells and reduced heme oxygenase-1 (HO-1) in the microenvironment, causing increased circulating CD8^+^ T cells and reduced T_reg_ cells, and resulting in enhanced immunosurveillance and clearance of tumor cells [41, 47]. Moreover, prolonged cycles of fasting have also been shown to protect immune cells during chemotherapeutic treatment [49, 50], suggesting the possibility of combining immunotherapies with traditional chemotherapy alongside such dietary interventions. Similarly, CR also maintains immunological fitness of CD4^+^ T cells during aging to enhance cancer immunotherapy, specifically OX40-agonist immunotherapy [50]. In addition, a CR regimen can reduce desmoplasia and the inflammatory microenvironment of tumors [9], previously shown to impede the delivery of therapeutic drugs to the tumor cells.

While preclinical studies are mounting on the effects of intermittent CR in combination with chemotherapy and radiation therapy, clinical studies are slow to follow, likely due to the concerns listed above. A summary of past and current clinical trials of intermittent CR, fasting-mimicking diets, and ketogenic diets in combination with anticancer therapies is included in Table 1. A small study comprising ten subjects diagnosed with malignancies, including breast, esophageal, prostate, and lung, who underwent a 48–140 h fast prior to and a 56 h post-chemotherapy fast revealed significant improvement in self-reported side effects of therapy, including nausea, vomiting, diarrhea, weakness, and fatigue [51, 52].

**Table 1:** List of ongoing or completed clinical trials including calorie restriction (CR) or CR mimetic diets or drugs in combination with chemotherapy or radiotherapy

Intervention	Drug treatment(s)	Disease(s)	Reference	Outcome/Anticipated completion date
Fasting-mimicking diet	Standard chemotherapy	Breast	NCT02126449 de Groot et al. 2015 [79]	December 2018
Fasting: up to 60 h	Taxanes	Prostate	NCT02710721	December 2017
Fasting: up to 48 h	Standard chemotherapy	Ovarian, breast	NCT01954836	Completed, no reported results
Fasting: 20–140 h	Docetaxel, paclitaxel, cyclophosphamide, carboplatin, gemcitabine, doxorubicin	Breast, esophagus, prostate, lung, uterine, ovarian	Safdie et al. 2009 NCT01304251 [52]	Reduced self-reported side effects
Fasting: up to 48 h	Gemcitabine, cisplatin	Primary and metastatic lesions	NCT00936364	September 2017
Ketogenic diet	Radiotherapy	Colorectal, breast	NCT02516501	June 2018
Ketogenic diet	Palliative chemotherapy	Glioblastoma	NCT02939378	December 2017
Ketogenic diet	Temozolomide	Glioblastoma	NCT02046187	Completed, no reported results
Ketogenic diet	Temozolomide, radiotherapy	Glioblastoma	NCT02302235	August 2016, recruiting
Ketogenic diet	Standard chemotherapy, radiotherapy	Glioblastoma	NCT03075514	March 2018
Intermittent energy restriction	Docetaxel, paclitaxel	Breast	B-AHEAD-2	Completed, no reported results
Everolimus	Docetaxel, cisplatin	Head and neck	NCT00935961	Completed, no reported results
Everolimus	Rituximab	B-cell lymphoma	NCT00790036	Completed, no reported results
Everolimus	Pemetrexed	Lung	NCT00434174	Completed, no reported results
Everolimus	Doxorubicin, bevacizumab	Breast	NCT02456857	Completed, no reported results
Everolimus	Bevacizumab, lapatinib	Breast	NCT00567554	Completed, no reported results
Everolimus	Cisplatin, navelbine, radiotherapy	Lung	NCT01167530	Completed, no reported results
Everolimus	Lenvatinib	Renal	NCT02915783	December 2018
Sirolimus	Etoposide, cyclophosphamide	Solid and central nervous system	NCT02574728	June 2020
Temsirolimus	Bevacizumab	Prostate	NCT01083368	Completed, no reported results
Temsirolimus	Standard AML chemotherapy	AML	NCT01611116	July 2017
Temsirolimus	Dexamethasone, mitoxantrone, vincristine, PEG-aspargase	AML, NHL	NCT01403415	Completed, no reported results
Metformin	Cisplatin, radiotherapy	Lung	NCT02115464	December 2017
Metformin	Anthracycline, taxane, platinum, capecitabine, vinorelbine	Metastatic breast	NCT01310231	March 2016; recruiting
Metformin	Radiation, carboplatin, paclitaxel	Lung	NCT02186847	March 2018
Metformin	Dexamethasone, mitoxantrone, vincristine, PEG-aspargase	ALL	NCT01324180	Completed, no reported results
Metformin	5-Fluorouracil	Colorectal	NCT01941953	Completed, no reported results
Metformin	Folfinic acid, oxaliplatin, leucovorin, 5-fluorouracil, irinotecan	Colorectal	NCT01926769	Completed, no reported results
Metformin	Cisplatin, radiotherapy	Head and neck	NCT02949700	December 2019
Metformin	Fluorouracil, doxorubicin, cyclophosphamide	Breast	NCT02506777	November 2015; recruiting
Metformin	Paclitaxel, carboplatin, docetaxel	Ovarian, fallopian tube, peritoneal	NCT02122185	February 2018
Metformin	Carboplatin, paclitaxel	Ovarian	NCT02312661	March 2017
Metformin	Gemcitabine, erlotinib	Pancreatic	NCT01210911	Completed, no reported results
Metformin	Standard chemotherapy	All tumor types	NCT01442870	Completed, no reported results
Hydroxycitrate	Folinic acid, oxaliplatin, leucovorin, 5-fluorouracil, irinotecan, cetuximab, vectibix, carboplatin, bevacizumab, cisplatin, gemcitabine	Metastatic lung, colon, ovarian, esophagus, uterine, liver, parotid, prostate	Schwartz et al. 2014 [68]	Hydroxycitrate is tolerated, but no added benefit was found

*AML* acute myeloid leukemia, *ALL* acute lymphoblastic leukemia, *NHL* non-Hodgkin’s lymphoma

Larger trials are currently underway to determine the potential for short-term fasting in reducing the side effects and efficacy of chemotherapies, and will likely be the launching point for future clinical trials with intermittent CR as a potential adjuvant therapy.

## CR mimetics

Given the nutritional concerns of CR and fasting in some cancer patients, CR mimetics, namely pharmacological agents that target pathways affected by CR, such as rapamycin, metformin, resveratrol, and hydroxycitrate, are attractive strategies to mimic the protective effects of CR both for cancer prevention and as adjuvant therapies without dietary restriction. These CR mimetics affect systemic and tumor-specific inflammation and metabolism, and targeting these pathways may sensitize cancers to traditional and emerging anti-cancer therapies by reducing tumor-associated inflammation or causing metabolic stress in the cancer cell.

Administration of the CR mimetic rapamycin (sirolimus), an immunosuppressant drug and established inhibitor of mTOR, extends lifespan and delays cancer in mice [53]. Our lab has shown that rapamycin or its analog, Afinitor® (everolimus), can mimic the anticancer effects of CR in mouse models of pancreatic and breast cancer [54, 55]. Signaling via the mTOR pathway has been implicated in a broad range of chemoresistant cancers [56], and rapamycin has been shown to reverse multidrug resistance [57]. Promising preclinical studies demonstrate that rapamycin can sensitize certain cancers to chemotherapy and radiation therapy. Active phase I and II clinical trials are ongoing to determine the effect of combining rapamycin with chemotherapeutic regimens, including gemcitabine for osteosarcoma, cyclophosphamide, dexamethasone in myeloma, and mitoxantrone, etoposide and cytarabine for leukemia.

Another CR mimetic, metformin, is a biguanide commonly used to treat type 2 diabetes by inhibiting gluconeogenesis through indirect activation of AMPK, thus reducing blood glucose and insulin to levels observed in CR mice [58]. Metformin, as a monotherapy, suppresses tumor development and/or growth in multiple experimental models, including colon, mammary, and hematopoietic cancer models [59]. Metformin has shown promise both in preclinical and clinical studies, improving treatment of colon, breast, ovarian, prostate, and lung cancers [60, 61]. Several phase II trials are currently underway to evaluate metformin as a potential combination therapy, including one non-small cell lung cancer study that involves a low carbohydrate diet arm.

Resveratrol, a polyphenolic compound found in grapes, berries and, most famously, red wine, has also been under consideration as a CR mimetic. Resveratrol is believed to underlie the “French paradox,” in which the consumption of red wine is believed to reduce mortality rates from cardiovascular disease and certain cancers [62]. Resveratrol displays anti-inflammatory, anti-oxidant, and anti-angiogenic [63] properties and suppresses development and growth of numerous cancer types in preclinical models, including breast, prostate, colon, and liver. Although several in vitro and in vivo studies have indicated that resveratrol can enhance anticancer treatments [64], Fukui et al. [65] suggested that resveratrol may actually reduce the efficacy of paclitaxel treatment in breast cancer. Therefore, more preclinical studies should be performed prior to progressing to clinical trials examining resveratrol as an adjuvant anticancer therapy.

An emerging CR mimetic is hydroxycitrate, a citric acid derivative and over-the-counter weight-loss drug that inhibits ATP citrate lyase, the enzyme that catalyzes the conversion of citrate into oxaloacetate and acetyl CoA. Cancer cells utilize acetyl CoA as a synthetic precursor to fuel proliferation and growth [66]; thus, blocking acetyl CoA synthesis is a rational approach to specifically target cancer metabolism. Furthermore, hydroxycitrate is a potent inducer of autophagy. Nevertheless, hydroxycitrate administration alone does not affect systemic glucose or insulin [67]. Further, although Pietrocola et al. [41] showed enhanced anticancer effects combining hydroxycitrate with doxorubicin and cyclophosphamide, a small trial revealed no added benefit of hydroxycitrate when administered with α-lipoic acid alongside the standard of care [68].

### Alternative dietary approaches

Further to the CR mimetic drugs above, dietary regimens, such as low carbohydrate/ketogenic and intermittent energy restriction (IER), may be suitable alternatives to chronic CR in combination therapies. Low carbohydrate/ketogenic diets rewire energy metabolism to utilize ketones derived from fatty acids, in particular medium chain triglycerides, as an energy source rather than glucose. These diets mimic many of the metabolic and anti-inflammatory properties of CR, including reduced blood glucose, insulin, and IGF-1 [69], as well as the oxidation of fatty acids and generation of ketones. The ketogenic diet has long been used successfully as a means to reduce epileptic seizures [70] and more recently in type 2 diabetes [71]. Further, studies have shown that the diet is well tolerated in cancer patients either as an adjuvant or monotherapy [72, 73]. Use as a monotherapy has been shown to halt progression of soft palate cancer [73], suggesting that, in some circumstances, a ketogenic diet alone may be sufficient for cancer management. Preclinical studies have shown promising results for low carbohydrate/ketogenic diets in reducing tumor growth in breast [74], prostate [75], brain [76], and gastric cancer models [77], and it has been shown to promote response to adjuvant radiation therapy [72]. In addition, a switch to a low carbohydrate/ketogenic diet has been shown to prevent cachexia in patients undergoing chemotherapy, suggesting this dietary approach may be a suitable alternative for cancer patients at risk of cachexia, sarcopenia, and weight loss [78].

IER, such as the 5–2 diet, in which an individual adheres to severe restriction (75% fewer calories) on 2 non-consecutive days while eating a normal, healthy diet on the remaining 5 days, has been a successful weight loss approach in human studies, and results in similar improvements in metabolic parameters such as insulin sensitivity [3]. A randomized trial is currently underway comparing IER and chronic CR in combination with taxane treatment in breast cancer patients. Preliminary results from this study suggest that IER is tolerable in patients receiving chemotherapy, and the outcome of this trial will add to the evidence for CR as a supportive treatment and evaluate IER as a feasible alternative to CR as an anticancer therapy.

## Conclusions

**Fig. 1:**
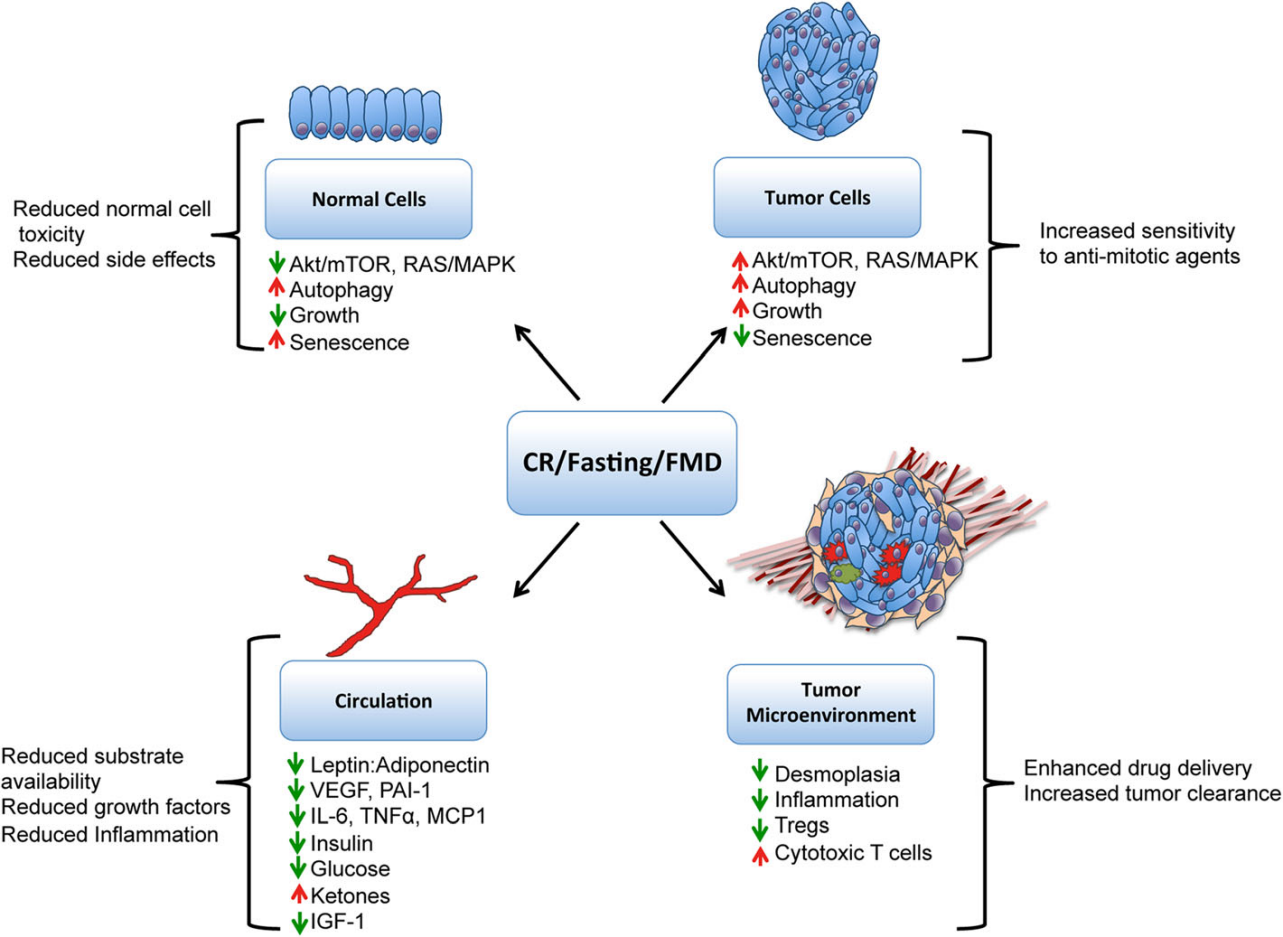
Mechanisms through which calorie restriction (CR) affects response to anticancer therapy. CR, fasting, or fasting-mimicking diets (FMDs) cause reduced Akt/mTOR and Ras signaling in normal cells, resulting in senescence, reduced growth, and protection from cytotoxic treatment, while in tumor cells, oncogenic signals remain and cells are sensitive to anti-mitotic therapies. CR, fasting, and FMD also reduce pro-inflammatory cytokines in the circulation and in the tumor microenvironment niche, as well as reduced leptin, insulin, IGF-1, and glucose. CR can reduce desmoplasia surrounding the tumor tissue, which may facilitate better therapeutic drug delivery to the tumor cells. CR can also aid in immunosurveillance of tumors by reducing T_reg_ populations that inhibit cytotoxic CD8^+^ T cells. This figure has not been published elsewhere

Dietary interventions are attractive as inexpensive supportive anticancer therapies. CR is an established tumor preventative regimen, reducing systemic inflammation and growth factor signaling, as well as improving metabolic markers. Improved metabolism and inflammation are also likely mechanisms through which CR may reduce tumor growth and enhance therapeutic response (Fig. 1). In addition, oncogenic transformation and loss of senescence in cancer cells may render them more sensitive to CR than normal cells (Fig. 1). As chronic CR is contraindicated for many cancer patients at risk for weight loss, cachexia, and immunosuppression, intermittent CR, fasting-mimicking diets, low carbohydrate/ketogenic diets, or CR mimetic drugs may be more suitable. Fasting and low carbohydrate diets have been shown to reduce side effects and to improve chemotherapy and radiation therapy in animal models, and there is great promise for these interventions in the clinic. More preclinical studies are required to determine in which cancers, at which stage, and in what combinations CR mimetic drugs may prove most effective. Future studies should take into consideration (1) the risk of cachexia in a patient population, whereby those at high risk may benefit from a ketogenic diet or short-term fasting; (2) the immunologic state of the enrolled patients, when CR or rapamycin treatment may be detrimental to wound healing or inflammatory responses; and (3) the metabolic state of patients, with diabetic patients in particular being at risk of adverse effects during chronic CR or fasting regimens, whereby treatment with metformin or a ketogenic diet may be of benefit. While in the short-term studies will need to focus on the safety and added benefit to current therapies, future studies may also focus on the potential of CR in enhancing the response to lower doses of chemotherapy and radiation therapy. In summary, CR and its mimetics show promise as supportive anticancer therapies. Clinical studies are ongoing and will inform on the potential use of these dietary and drug treatments alongside conventional treatments.

## Abbreviations

AMPK: AMP kinase
CR: Calorie restriction
IER: Intermittent energy restriction
IGF-1: Insulin-like growth factor 1
